# Singlet oxygen production by photosystem II is caused by misses of the oxygen evolving complex

**DOI:** 10.1111/nph.18514

**Published:** 2022-10-14

**Authors:** Heta Mattila, Sujata Mishra, Taina Tyystjärvi, Esa Tyystjärvi

**Affiliations:** ^1^ Department of Life Technologies/Molecular Plant Biology University of Turku FI‐20014 Turku Finland

**Keywords:** anaerobic, anoxia, cyanobacteria, *F*_V_/*F*_M_, histidine, photodamage, reactive oxygen species

## Abstract

- Singlet oxygen (^1^O_2_) is a harmful species that functions also as a signaling molecule. In chloroplasts, ^1^O_2_ is produced via charge recombination reactions in photosystem II, but which recombination pathway(s) produce triplet Chl and ^1^O_2_ remains open. Furthermore, the role of ^1^O_2_ in photoinhibition is not clear.
- We compared temperature dependences of ^1^O_2_ production, photoinhibition, and recombination pathways.
- ^1^O_2_ production by pumpkin thylakoids increased from −2 to +35°C, ruling out recombination of the primary charge pair as a main contributor. S_2_Q_A_^−^ or S_2_Q_B_^−^ recombination pathways, in turn, had too steep temperature dependences. Instead, the temperature dependence of ^1^O_2_ production matched that of misses (failures of the oxygen (O_2_) evolving complex to advance an S‐state). Photoinhibition *in vitro* and *in vivo* (also in *Synechocystis*), and in the presence or absence of O_2_, had the same temperature dependence, but ultraviolet (UV)‐radiation‐caused photoinhibition showed a weaker temperature response.
- We suggest that the miss‐associated recombination of P_680_^+^Q_A_^−^ is the main producer of ^1^O_2_. Our results indicate three parallel photoinhibition mechanisms. The manganese mechanism dominates in UV radiation but also functions in white light. Mechanisms that depend on light absorption by Chls, having ^1^O_2_ or long‐lived P_680_^+^ as damaging agents, dominate in red light.

## Introduction

Molecular oxygen (O_2_) in its ground state is a biradical, as the two unpaired electrons have parallel spins (thus, O_2_ is a triplet). Exchange of energy and spin with a molecule in a triplet excited state can turn O_2_ to a highly reactive singlet state (^1^O_2_) – for a review, see Schweitzer & Schmidt (2003). ^1^O_2_ causes cellular damage by oxidizing biomolecules containing double bonds (Schweitzer & Schmidt, 2003; Halliwell & Gutteridge, 2015; Di Mascio *et al*., 2019). ^1^O_2_ also generates cellular signals leading to acclimation to high light and/or to cell death via apoptotic pathways in photosynthetic organisms (Lee *et al*., 2007; Ramel *et al*., 2012b; Crawford *et al*., 2018).

Both photosystem I (PSI) and photosystem II (PSII) produce ^1^O_2_ (Macpherson *et al*., 1993; Cazzaniga *et al*., 2012), the majority being produced by the PSII core after a charge recombination reaction (Telfer *et al*., 1994; Ramel *et al*., 2012a); however, contrasting data have also been published (Santabarbara *et al*., 2007). The excited triplet state of the PSII reaction center Chl(s) (^3^P_680_) can react with the ground‐state O_2_, which results in the formation of ^1^O_2_ and the singlet ground state of P_680_. ^3^P_680_ can be produced by the recombination of the charge pairs S_2_Q_A_^−^, S_3_Q_A_^−^, S_2_Q_B_^−^, and S_3_Q_B_^−^, by the recombination of the primary charge pair P_680_^+^Pheo^−^, or by the recombination of P_680_^+^Q_A_^−^. The recombination reactions S_2/3_Q_A/B_^−^ → S_1/2_Q_A/B_ are rare, with time constant of *c*. 1 s for S_2_Q_A_^−^ and 20–30 s for S_2_Q_B_^−^ (Rutherford, 1989; Tyystjärvi & Vass, 2004), but have been suggested to be responsible for ^1^O_2_ production in weak light (Keren *et al*., 1997). The primary pair, in turn, recombines with nanosecond kinetics and may produce ^3^P_680_ when Q_A_ is reduced (Vass & Styring, 1993). The P_680_^+^Q_A_^−^ pair is available for recombination only if the O_2_‐evolving complex (OEC) of PSII fails to reduce P_680_^+^.

A transient failure of the OEC to reduce P_680_^+^ is called a miss, and *c*. 8% of charge separations fail in this way (Forbush *et al*., 1971; Isgandarova *et al*., 2003; Pham *et al*., 2019). In this case, charge separation leads to reduction of Q_A_, followed by recombination of P_680_^+^Q_A_^−^. Misses represent reaction equilibria between S‐state advancement and recombination reaction in normal, active PSII. The percentage of misses is similar in the thermophilic cyanobacterium *Cyanosiphon merolae* and in spinach, although the redox potential of the Q_A_/Q_A_^−^ pair is much less negative in *C. merolae* than in spinach (Pham *et al*., 2019). Furthermore, the time constant of the recombination of P_680_^+^Q_A_^−^ is 100–200 μs in Tris‐washed thylakoids, which lack a functional donor side (Renger & Wolff, 1976); the kinetics in active PSII are not known. A 100–200 μs time constant of the P_680_^+^Q_A_^−^ recombination reaction would make misses highly competitive against the majority of S‐state transitions. These considerations imply that a miss occurs because the rate of S‐state advancement in a fraction of OECs is so slow that P_680_^+^Q_A_^−^ recombines, not because the recombination occasionally wins competition with normal advancement of the S‐state. Electron paramagnetic resonance (EPR) measurements (Han *et al*., 2012) and modeling of flash O_2_ data (Pham *et al*., 2019) indicate that most misses occur in the S_2_ to S_3_ transitions, but other S‐states may also fail to advance.

It has been suggested that ^1^O_2_ is responsible for photoinhibition of PSII – for reviews, see Vass (2012), Tyystjärvi (2013), and Zavafer & Mancilla (2021) – as positive shifts in the redox potentials of PSII electron acceptors limit ^1^O_2_ production and protect against photoinhibition (Fufezan *et al*., 2002, 2007; Davis *et al*., 2016; Treves *et al*., 2016) and because PSII can be protected by carotenoids that quench ^1^O_2_ (Jahns *et al*., 2000; Hakkila *et al*., 2013). However, photoinhibition is known to occur under ultraviolet (UV) radiation (Jones & Kok, 1966; Hakala *et al*., 2005) and in anaerobic conditions (Nedbal *et al*., 1992; Sundby *et al*., 1992) where ^1^O_2_ is not formed (Hideg *et al*., 1994), indicating that either ^1^O_2_ does not cause photoinhibition or photoinhibition has alternative or parallel mechanisms. In addition, ^1^O_2_ is known to slow down the PSII repair cycle, as it damages oxidation‐prone translation factors, reducing the overall translation efficiency (Nishiyama *et al*., 2004).

To better understand ^1^O_2_ production and photoinhibition, we measured their temperature dependences and compared them with temperature dependences of recombination pathways. The results indicate that the miss‐associated recombination reaction is crucial for the formation of ^1^O_2_. The temperature dependence of photoinhibition, in turn, was similar but not identical under different wavelengths, suggesting that several mechanisms contribute to photoinhibition. In white light, the contribution of the manganese (Mn) mechanism (Hakala *et al*., 2005) was estimated to be 63–68%. Under monochromatic light, contributions from two other mechanisms (a ^1^O_2_‐dependent and a P_680_^+^‐dependent mechanism) increase toward longer wavelengths.

## Materials and Methods

### Growth conditions

Pumpkin (*Cucurbita maxima* L.) was grown at 20°C, in a 16 h light period, with photosynthetic photon flux density (PPFD) 150–200 μmol m^−2^ s^−1^. Thylakoid membranes were isolated as previously described (Hakala *et al*., 2005) and stored at −75°C in a storage buffer (10 mM HEPES (pH 7.4), 0.5 M sorbitol, 10 mM magnesium chloride (MgCl_2_), and 5 mM sodium chloride (NaCl)). *Synechocystis* sp. PCC 6803 cells were maintained on BG‐11 agar plates (Rippka *et al*., 1979) supplied with 20 mM HEPES–sodium hydroxide (NaOH) (pH 7.5), under continuous light (PPFD 40 μmol m^−2^ s^−1^) at 32°C. A few days before the experiments, *Synechocystis* cells were transferred to liquid cultures with mixing, otherwise under similar conditions.

### Singlet oxygen measurements

Pumpkin thylakoids (100 μg Chl ml^−1^) were incubated for 5 min in a photoinhibition buffer (40 mM HEPES–potassium hydroxide (pH 7.4), 1 M betaine monohydrate, 330 mM sorbitol, 5 mM MgCl_2_, and 5 mM NaCl) in darkness and then illuminated for 2 min with strong light, and the light‐induced changes in O_2_ concentration in the absence and presence of 20 mM l‐histidine (Sigma‐Aldrich) were recorded (Telfer *et al*., 1994; Rehman *et al*., 2013). An optode (Firesting O_2_ FSO2‐0x with OXSP5 sensor spots; PyroScience GmbH, Aachen, Germany), a homemade cuvette, and a 10 W cold‐white LED (PPFD 2000 μmol m^−2^ s^−1^; for the spectrum, see Supporting Information Fig. S1) were used for measurements at 5, 15, 25, and 35°C. The optode was calibrated with air‐saturated water. A measurement at −2°C, where water cannot be used for calibration, and a comparison measurement at 15°C, were done using an O_2_ electrode (Hansatech, King's Lynn, UK) and a slide projector equipped with a halogen lamp (PPFD 3000 μmol m^−2^ s^−1^; for the spectrum, see Fig. S1). The O_2_ electrode was calibrated with air‐saturated photoinhibition buffer that remained liquid at −2°C, before and after addition of solid sodium dithionite to remove O_2_. With both devices, the rate of ^1^O_2_ production was calculated as the difference in the rate of O_2_ consumption in the presence and absence of 20 mM histidine. The temperature dependence of the reaction between histidine and ^1^O_2_ was tested by illuminating (PPFD 2000 μmol m^−2^ s^−1^) 1 μM rose bengal in the presence of 20 mM histidine. No significant consumption of O_2_ was observed in the absence of histidine.

### Recombination reactions

Thermoluminescence was measured with a homemade luminometer from pumpkin thylakoids (600 μg Chl ml^−1^) as previously described (Tyystjärvi *et al*., 2009), in the presence and absence of 20 μM 3‐(3,4‐dichlorophenyl)‐1,1‐dimethylurea (DCMU), with a heating rate of 0.56°C s^−1^. A 1 J xenon pulse was fired at −10°C. The rate constants of the S_2/3_Q_A/B_^−^ recombination reactions were calculated as functions of temperature with Copasi (Hoops *et al*., 2006) assuming that each rate constant depends on temperature according to the Arrhenius equation. Three competing recombination routes (direct, indirect, and excitonic) were assumed for the analysis of the Q band (Rappaport & Lavergne, 2009), whereas the B band was analyzed as a single reaction (Randall & Wilkins, 1945; Tyystjärvi & Vass, 2004).

### Fluorescence measurements in the light

Fluorescence parameters were measured during illumination from *Synechocystis* cells (optical density at 730 nm 0.8–1.1), pumpkin thylakoids (100 μg Chl ml^−1^), and detached pumpkin leaves with a PAM‐2000 (Walz, Effeltrich, Germany) at 5–35°C. A saturating pulse was fired to calculate (*F*_M_ − *F*_0_)/*F*_M_ (=*F*_V_/*F*_M_) after dark acclimation (30 s for thylakoids and 30 min for leaves). Thereafter, the sample was illuminated with white light (PPFD 750 μmol m^−2^ s^−1^ from a slide projector for *Synechocystis*, and PPFD 1500 μmol m^−2^ s^−1^ from a 500 W high‐pressure xenon lamp with a water filter for pumpkin; for the spectra, see Fig. S1). To calculate (*F*_M_′ − *F*)/*F*_M_′, saturating pulses were fired after 1 min for thylakoids and after 15, 30, and 45 min for *Synechocystis* cells and pumpkin leaves. To estimate the reduction state of *Q*_A_, 1 − qL (Kramer *et al*., 2004) was calculated, using an estimation (Oxborough & Baker, 1997) for *F*_0_′ (see Mattila *et al*., 2020).

### Photoinhibition treatments

Pumpkin thylakoids (100 μg Chl ml^−1^), detached pumpkin leaves, or intact *Synechocystis* cells (optical density at 730 nm 0.8–1.1) were illuminated at various PPFD values, wavelengths, and temperatures, as indicated. Before a treatment, leaves were incubated overnight at PPFD 10–20 μmol m^−2^ s^−1^ with the petioles in a solution with 0.4 mg ml^−1^ lincomycin (Sigma‐Aldrich). Lincomycin (0.4 mg ml^−1^) was added to *Synechocystis* cells right before the treatment. Thylakoids were illuminated in photoinhibition buffer, unless otherwise mentioned, and *Synechocystis* cells in BG‐11. The samples were mixed during the treatments. Red (> 650 nm) or blue (400–450 nm) light was obtained with a 500 W high‐pressure xenon lamp equipped with a long‐pass or a short‐pass edge filter (LL‐650 and LS‐450, respectively; Corion, Holliston, MA, USA). Monochromatic light was obtained with band‐pass filters (full width at half maximum 10 nm; Corion; Newport, Irvine, CA, USA). UV radiation was obtained with VL‐8.LC (365 and 254 nm) and VL‐8.M (312 nm) lamps (Vilber Lourmat, Collégien, France; for the spectra, see Havurinne *et al*., 2021). White light was obtained from a 1000 W (Sciencetech, London, ON, Canada) or 500 W (Oriel Instruments; Newport) high‐pressure xenon lamp (when measuring the temperature dependence of photoinhibition in thylakoids at 5°C intervals, and for the temperature dependence of photoinhibition in leaves), from a slide projector equipped with a low‐voltage halogen lamp (*Synechocystis*), or from a 10 W cold‐white LED (all other experiments). For laser‐pulse‐induced photoinhibition, pumpkin thylakoids (27 μl, 76 μg Chl ml^−1^) were illuminated in a 3 × 3 × 10 mm^3^ cuvette with 532 nm, 4 ns, 12.5 mJ pulses from a Nd : YAG laser (Continuum, San Jose, CA, USA). The interval between the laser pulses was 0.1 s (240 flashes in total), 10 s (100 flashes) or 30 s (40 flashes).

α‐Tocopherol, when used, was vigorously mixed in dimethyl sulfoxide and immediately added to thylakoid suspension, which was then vigorously mixed for 20 s. In control experiments, only dimethyl sulfoxide was added. Anaerobic conditions, applied when indicated, were achieved by flushing the sample continuously with nitrogen (N_2_) gas. In control (aerobic) experiments, the sample was flushed with air. Freshly prepared 20 mM sodium bicarbonate was used to test recovery of PSII activity in isolated thylakoids after anaerobic photoinhibition.

Experiments with isolated thylakoids were always repeated under otherwise identical conditions in the dark, to determine the rate of dark inactivation of PSII.

### Quantification of photoinhibition

Before and after treatments, light‐saturated O_2_ evolution was measured from aliquots of treated thylakoids or from thylakoids isolated from treated leaves, at 22°C, or from aliquots of illuminated *Synechocystis* suspension at 32°C, using an O_2_ electrode (Hansatech, King's Lynn, UK) as previously described (Hakala *et al*., 2005) with artificial electron acceptors (0.5 mM 2,6‐dimethylbenzoquinone (DMBQ) with thylakoids; 0.5 mM 2,6‐dichlorobenzoquinone and 0.5 mM hexacyanoferrate(III) with *Synechocystis*). In some experiments, as indicated, PSII activity was estimated by measuring the fluorescence parameter *F*_V_/*F*_M_ with a Fluorpen (Photon Systems Instruments, Brno, Czech Republic) after at least 5 min (thylakoids) or 30 min (leaves) dark incubation.

The rate constant of photoinhibition *k*_PI_ was calculated by fitting the loss of O_2_ evolution or decrease in *F*_V_/*F*_M_, as indicated, to the first‐order reaction equation in Sigmaplot (Systat Software Inc., Palo Alto, CA, USA). In the case of thylakoids, the final *k*_PI_ values were obtained by subtracting the first‐order rate constant of dark inactivation from the raw *k*_PI_ value.

### Activation energy

The activation energy *E*_a_ was calculated by fitting the dependence of the rate constant *k* on absolute temperature *T* to the Arrhenius equation by using linear regression of log_e_(*k*) to 1/*T* according to the equation log_e_(*k*) = −*E*_a_/(*k*_b_ *T*) + constant, where *k*_b_ is Boltzmann's constant.

### Detection of carbon‐centered radicals

We mixed 5.9 mg of α‐(4‐pyridyl 1‐oxide)‐*N*‐*tert*‐butylnitrone (POBN; Enzo Life Sciences Inc., New York, NY, USA) in 0.6 ml of thylakoid suspension (100 μg Chl ml^−1^) to get a final concentration of 50 mM POBN. POBN‐R‐adduct (the reaction product of POBN and a carbon (C)‐centered radical) was detected before and immediately after illumination or dark incubation with an EPR spectrometer (Miniscope MS 5000; Magnettech GmbH, Berlin, Germany). The measurement parameters were as follows: 60 s sweep time (three technical repetitions) at 330–340 mT, 0.2 mT modulation, 100 kHz frequency, and 10 mW power. C‐centered radicals were quantified by the height of the first positive peak at 334.5–334.8 mT of the EPR signal.

## Results

### Singlet oxygen production by thylakoid membranes shows a positive temperature dependence

**Fig. 1:**
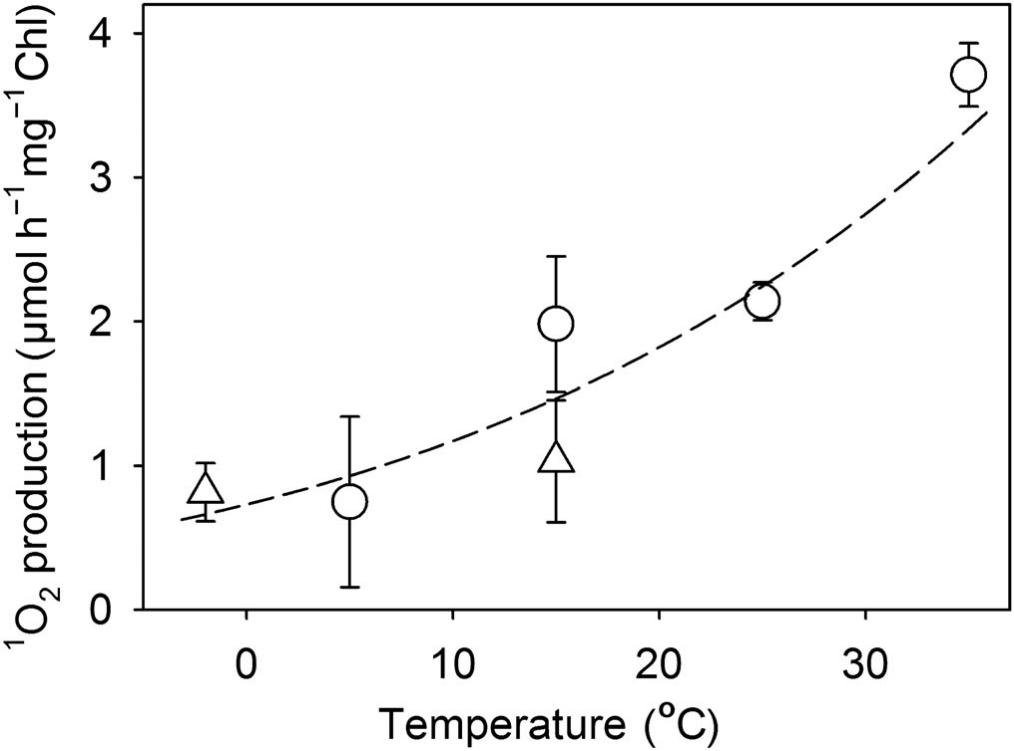
Temperature dependence of singlet oxygen (^1^O_2_) production under intense white light by isolated pumpkin thylakoids, measured with a histidine‐based method. Oxygen (O_2_) was measured with an optode (circles) or with an O_2_ electrode (triangles). The measurement device was calibrated in water (circles) or in the photoinhibition buffer (triangles). The data measured at photosynthetic photon flux density (PPFD) 3000 μmol m^−2^ s^−1^ (triangles) have been normalized to PPFD 2000 μmol m^−2^ s^−1^ by multiplying by 2/3. Each data point represents an average of at least three independent measurements, and the error bars show SDs. The dashed line shows the best fit to the Arrhenius equation, revealing an *E*_a_ of 0.31 eV.

Isolated pumpkin thylakoids were illuminated in high light at −2, 5, 15, 25, and 35°C, and ^1^O_2_ production during the illumination was measured with the histidine method (Rehman *et al*., 2013). ^1^O_2_ production increased five‐fold from −2 to +35°C, and the data showed a reasonable fit to the Arrhenius equation (Fig. 1). The reaction between ^1^O_2_ and histidine, probed by using rose bengal as a ^1^O_2_ sensitizer, showed much weaker temperature dependence (Fig. S2), indicating that the temperature dependence in Fig. 1 reflects the temperature dependence of ^1^O_2_ production by thylakoids and not that of the reaction between ^1^O_2_ and histidine.

### Temperature dependence of singlet oxygen production matches that of the misses

Photosystem II is responsible for most ^1^O_2_ produced by thylakoids (Cazzaniga *et al*., 2012). However, to understand which PSII charge recombination reaction is responsible for the ^1^O_2_ production, the temperature dependences of recombination reactions were compared with the observed temperature dependence of ^1^O_2_ formation. The sub‐nanosecond recombination of P_680_^+^Pheo^−^ is known to occur readily even at 77 K (Zabelin *et al*., 2016), indicating negligible *E*_a_, and the triplet state ^3^P_680_ has only *c*. 0.025 eV lower energy than the P_680_^+^Pheo^−^ radical pair (Dau & Zaharieva, 2009), implying that the temperature dependence of triplet formation via recombination right after primary charge separation is negligible at physiological temperatures. Therefore, rapid recombination of the primary pair does not account for the observed ^1^O_2_ formation by PSII.

Next, recombination of the charge pairs consisting of a reduced quinone acceptor (Q_A_^−^ or Q_B_^−^) and a hole in the OEC in the state S_2_ were studied with thermolumiscence. The rate constants of the S_2_Q_A_^−^ → S_1_Q_A_ and S_2_Q_B_^−^ → S_1_Q_B_ recombination reactions can be calculated from the thermoluminescence Q (with DCMU) and B (no DCMU) bands, respectively. Previously, it has been shown that three competing pathways operate for the S_2_Q_A_^−^ → S_1_Q_A_ recombination (Rappaport & Lavergne, 2009). The ‘excitonic’ pathway leads, via P_680_^+^Pheo^−^, to the short‐lived singlet excited state of P_680_ and produces the luminescence. The ‘indirect’ pathway also has the primary pair as an intermediate; it does not produce luminescence but can instead lead to the formation of ^3^P_680_ when the P_680_^+^Pheo^−^ pair recombines. The third, also non‐luminescent, pathway has been interpreted as direct recombination of a hole in the OEC and an electron in Q_A_^−^ without the primary pair as an intermediate (Rappaport & Lavergne, 2009).

**Fig. 2:**
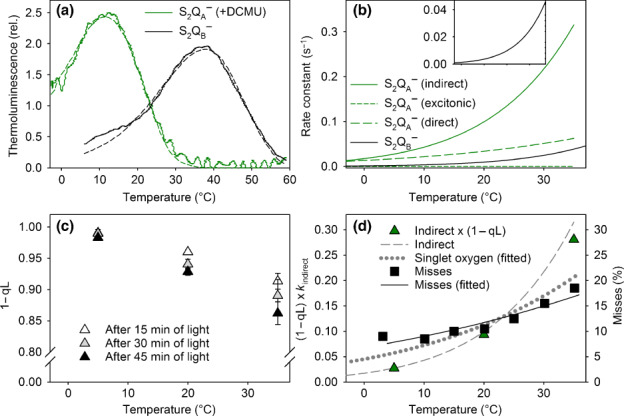
Temperature dependences of charge recombination reactions and reduction state of Q_A_. (a) Thermoluminescence Q (solid green line; in the presence of 3‐(3,4‐dichlorophenyl)‐1,1‐dimethylurea (DCMU)) and B (solid black line) bands were measured from isolated pumpkin thylakoid membranes after a xenon flash at −10°C. The underlying dashed lines show the best fits to a three‐reaction model (Rappaport & Lavergne, 2009) for the Q band, and to the Randall & Wilkins (1945) model for the B band. Each experimental curve represents an average of three independent measurements. (b) Temperature dependences of the rate constants of the three routes of S_2_Q_A_^−^ recombination (green continuous and dashed lines) and the S_2_Q_B_^−^ recombination (black continuous line), calculated based on the thermoluminescence data in (a). The inset shows S_2_Q_B_^−^ recombination in a narrower *y*‐axis scale. (c) Temperature dependence of the fraction of closed photosystem II centers (Q_A_^−^), measured as the 1 − qL Chl *a* fluorescence parameter in pumpkin leaves after 15 min (white triangles), 30 min (light gray triangles), or 45 min (black triangles) illumination with white light (photosynthetic photon flux density 1500 μmol m^−2^ s^−1^). Each data point represents an average of at least three independent measurements, and the error bars show SDs. (d) The rate constant of the indirect recombination (*k*_indirect_) from (b) multiplied by (1 – qL) at 30 min from (c) (green triangles), and the temperature dependence of misses in spinach thylakoids (black squares), measured by Isgandarova *et al*. (2003). The underlying black line shows the best fit to the Arrhenius equation. The gray dashed line shows the temperature dependence of *k*_indirect_ from (b), and the gray dotted line shows singlet oxygen production from Fig. 1, normalized to have the same intersection as the other curves, for comparison.

The Q band peaked at 12°C and the B band at 37°C (Fig. 2a). We applied the model of Rappaport & Lavergne (2009) for the analysis of the Q band (see also Rantamäki & Tyystjärvi, 2011). For the B band, a first‐order model with one recombining component (Randall & Wilkins, 1945; Tyystjärvi & Vass, 2004) was used. The rate constant of each pathway was obtained by fitting the curve to the respective model (Table S1). The rate constant of the indirect pathway of S_2_Q_A_^−^ recombination (*k*_indirect_) and the rate constant of S_2_Q_B_^−^ recombination (the reactions that may lead to ^1^O_2_ production) showed steep temperature dependences in the −2 to +35°C range (Fig. 2b). Such direct comparisons between the rate constant of recombination and ^1^O_2_ formation are justified in isolated thylakoids in which PSII reaction centers would remain essentially closed during illumination, irrespective of the temperature (Fig. S3). In leaves, however, the rate of ^3^P_680_ production from a recombination reaction under continuous illumination would be proportional to the rate constant of the recombination times the concentration of its substrate (a reduced quinone in this case). To measure the effect of temperature on the closure of PSII centers, we estimated the relative concentration of Q_A_^−^, [Q_A_^−^]_rel_, at 5, 20, and 35°C, in pumpkin leaves using the fluorescence parameter 1 − qL (Figs 2c, S3). However, the product *k*_indirect_ × [Q_A_^−^]_rel_ showed a highly similar temperature dependence to the rate constant k_indirect_ alone, much steeper than the temperature dependence of ^1^O_2_ production (Fig. 2d). The temperature dependence of the rate constant of S_2_Q_B_^−^ recombination (inset of Fig. 2b) was also steeper than that of ^1^O_2_ formation; no correction for the *in vivo* rate of S_2_Q_B_^−^ recombination was deemed necessary because Q_B_ is a two‐electron carrier and therefore the concentration of Q_B_^−^ would not depend strongly on temperature in continuous light. Thermoluminescence peaks originating from the recombination of the S_3_Q_A_^−^ and S_3_Q_B_^−^ states are not drastically different from those related to the S_2_ state (Vass & Govindjee, 1996); recombination reactions involving the S_3_ state instead of S_2_ would therefore not change the conclusions drawn herein. The ‘direct’ pathway of S_2_Q_A_^−^ recombination also contributed to our experimental data (Fig. 2b), but this pathway does not have a radical pair intermediate (Rappaport & Lavergne, 2009) and therefore cannot contribute to ^1^O_2_ production. To summarize, the S_2/3_Q_A/B_^−^ → S_1/2_Q_A/B_ recombination reactions cannot be the main producers of ^1^O_2_ in thylakoids.

The temperature dependence of misses of OEC has been earlier measured from spinach thylakoids (Isgandarova *et al*., 2003), and a comparison shows that the temperature dependence of misses, especially at 10–35°C, is highly similar to that of ^1^O_2_ production (Figs 1, 2). These data suggest that ^1^O_2_ is produced mainly via the miss‐associated P_680_^+^Q_A_^−^ recombination reaction (see Discussion section for details).

### Temperature dependence of photoinhibition is positive, universal among different species, and depends on the wavelength of illumination

**Fig. 3:**
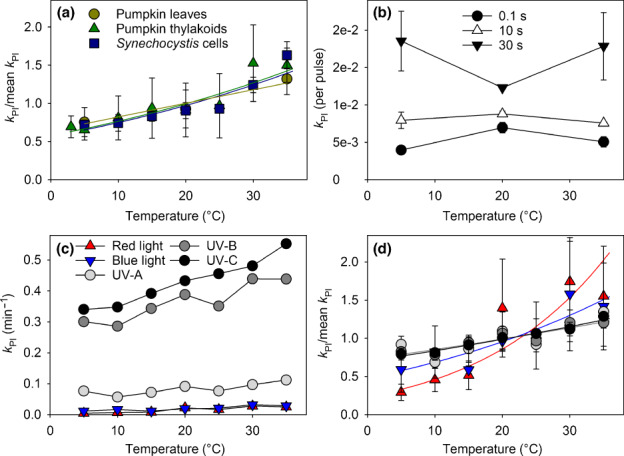
Temperature dependence of photoinhibition. (a) Temperature dependences of rate constants of photoinhibition *k*_PI_ in lincomycin‐treated pumpkin leaves (dark yellow circles), pumpkin thylakoids (green triangles), and lincomycin‐treated *Synechocystis* cells (blue squares). Photoinhibition was caused by illumination with white light (photosynthetic photon flux density (PPFD) 1500 μmol m^−2^ s^−1^ for thylakoids and leaves, PPFD 750 μmol m^−2^ s^−1^ for *Synechocystis*). Symbols represent experimental data, and the underlying lines show the best fits to the Arrhenius equation. (b) Temperature dependence of photoinhibition induced by 4 ns, 532 nm laser pulses fired with intervals of 0.1 s (closed circles), 10 s (open triangles), or 30 s (closed triangles) in pumpkin thylakoids. (c, d) Temperature dependence of photoinhibition induced by red (red upward triangles) or blue (blue downward triangles) light (PPFD 1500 μmol m^−2^ s^−1^), and by ultraviolet (UV)‐A (light gray circles; PFD 300 μmol m^−2^ s^−1^), UV‐B (dark gray circles; PFD 500 μmol m^−2^ s^−1^), or UV‐C (black circles; PFD 300 μmol m^−2^ s^−1^) radiation in pumpkin thylakoids. (d) Symbols represent experimental data, and the underlying lines show the best fits to the Arrhenius equation. The *k*_PI_ values were calculated by fitting the light‐induced decline in the rate of light‐saturated oxygen evolution of photosystem II (water to an artificial electron acceptor) to the first‐order reaction equation, individually for each experiment. The symbols show average *k*_PI_ values, based on at least three measurements, and error bars, drawn if larger than the symbol, show SDs, except for (b) where fitting was done on the averaged data; error bars show the SE of the fit. See Supporting Information Fig. S4 for details. In (a) and (d), the *k*_PI_ values have been normalized to their respective average values to facilitate comparison.

Connections between ^1^O_2_ and photoinhibition observed in previous literature – for a review, see Tyystjärvi (2013) – prompted us to compare their temperature dependences. The temperature dependence of photoinhibition was measured by illuminating isolated pumpkin thylakoids with strong white light at 3–35°C. The loss of light‐saturated O_2_ evolution (water (H_2_O) to DMBQ), measured from aliquots of the treated suspension, was fitted to the first‐order reaction equation (Fig. S4a) to obtain a raw rate constant, from which the rate constant of dark inactivation, occurring in isolated thylakoids, was subtracted to calculate the rate constant of photoinhibition *k*_PI_ (Fig. S4b). To ensure that the results are not a property of isolated systems only, the temperature dependence of photoinhibition was also measured *in vivo* by illuminating intact pumpkin leaves or cells of the cyanobacterium *Synechocystis* sp. PCC 6803 in the presence of lincomycin to block PSII repair (Fig. S4c). The data show an essentially identical, positive temperature dependence of photoinhibition for pumpkin thylakoids, *Synechocystis* cells, and pumpkin leaves; *k*_PI_ approximately doubled in the measured physiological temperature range (Fig. 3a). Furthermore, the temperature dependence of photoinhibition resembled those of misses and ^1^O_2_ production (Fig. 2d).

Photoinhibition by strong nanosecond laser pulses has earlier been suggested to be caused by ^1^O_2_ specifically originating from S_2_Q_A_^−^ and S_2_Q_B_^−^ recombination reactions (Keren *et al*., 1997). Measurements of laser‐pulse‐induced photoinhibition in pumpkin thylakoids (Fig. 3b) confirmed the characteristic dependence of the photoinhibitory efficiency (per flash) on the time interval between the flashes (Keren *et al*., 1997). However, comparison of Figs 1, 2, 3 shows that the temperature response of the laser‐pulse‐induced photoinhibition does not resemble the temperature dependence of any of the S_2_Q_A_^−^ or S_2_Q_B_^−^ recombination pathways, indicating that photoinhibition induced with short laser pulses is not related to these recombination reactions. In addition, the temperature dependence of laser‐pulse‐induced photoinhibition did not resemble that of photoinhibition induced by continuous light (Fig. 3a), suggesting a different photoinhibitory mechanism.

The resemblance of the temperature dependence of photoinhibition caused by continuous high‐intensity white light (Fig. 3a) with those of misses and ^1^O_2_ production (Fig. 2d) suggests, in turn, that the miss‐associated recombination reaction leads to the production of ^1^O_2_, which then damages PSII. As ^1^O_2_ is not produced under UV radiation (Hideg & Vass, 1996), we tested whether the temperature dependence of photoinhibition would be lost in UV radiation. Different wavelengths of visible light were also tested. As shown previously (e.g. Hakala *et al*., 2005), the *k*_PI_ value, when compared with photon flux density, is much higher under UV radiation than under visible light (Fig. 3c). Normalized data show that the positive temperature dependence remains, although it is somewhat milder in UV than in visible wavelengths, especially than in red light (Fig. 3d).

### Photoinhibition proceeds similarly under aerobic and anaerobic conditions

**Fig. 4:**
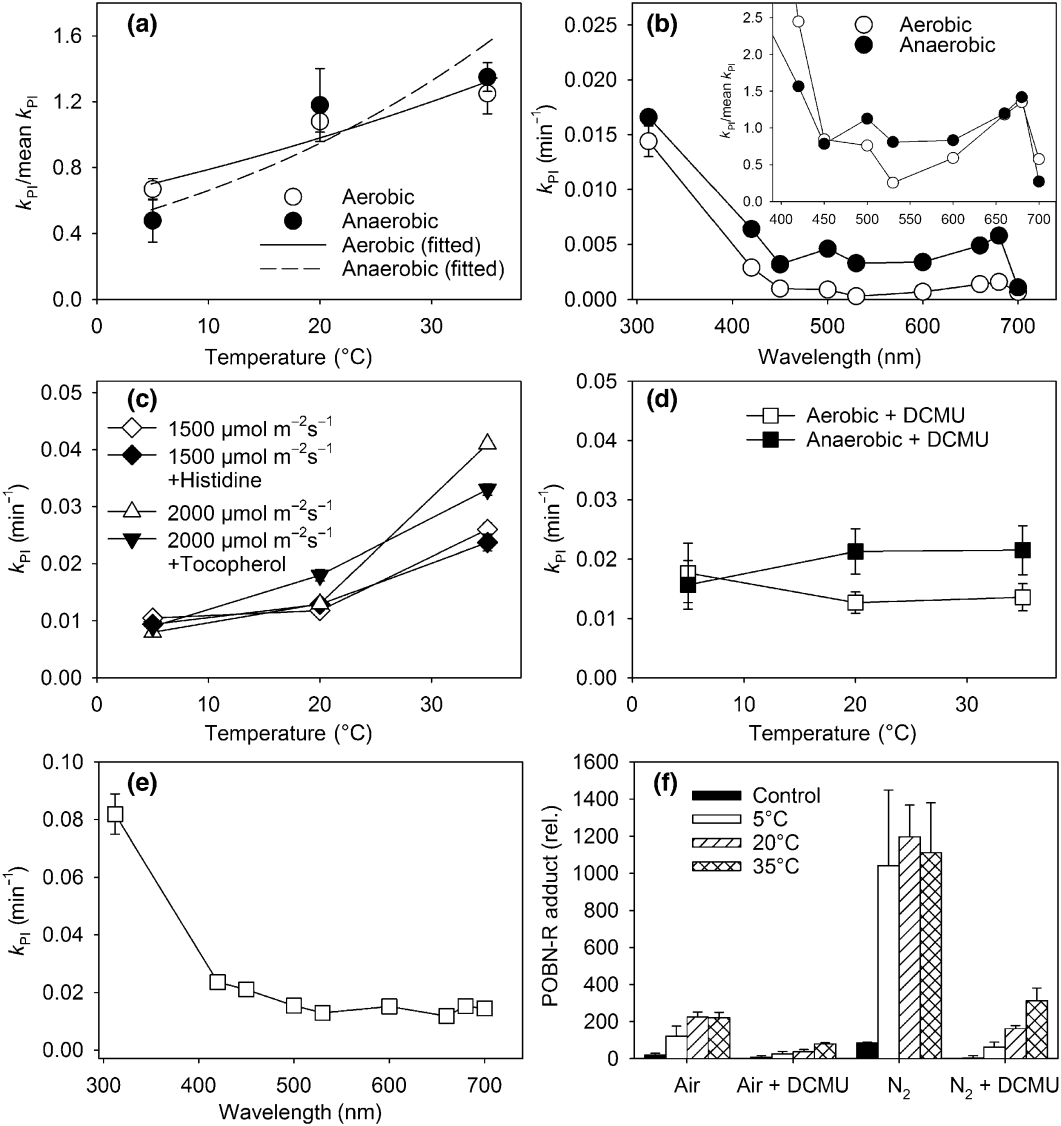
Photoinhibition in pumpkin thylakoids under anaerobic conditions, in the presence of 3‐(3,4‐dichlorophenyl)‐1,1‐dimethylurea (DCMU) and quenchers of singlet oxygen (O_2_). (a) Temperature dependence and (b) action spectrum of photoinhibition under aerobic (constant air bubbling; open circles) and anaerobic (constant nitrogen (N_2_) bubbling; closed circles) conditions. In (a) and in the inset showing only the visible wavelength range in (b), the rate constants of photoinhibition *k*_PI_ have been normalized to their respective mean values. The photosynthetic photon flux densities (PPFDs) were (a) 1150 μmol m^−2^ s^−1^ of white light and (b) 400 μmol m^−2^ s^−1^ at indicated wavelengths. (c) Temperature dependence of photoinhibition induced with white light (PPFD 2000 or 1500 μmol m^−2^ s^−1^, as indicated) under aerobic conditions in the absence (open diamonds) or presence of 5 mM histidine (closed diamonds), and in the absence (open upward triangles) or presence of 0.5 mM α‐tocopherol (closed downward triangles). (d) Temperature dependence and (e) action spectrum of photoinhibition in the presence of DCMU under aerobic (constant air bubbling; open squares) and anaerobic (constant N_2_ bubbling; closed squares) conditions at (d) PPFD 1150 μmol m^−2^ s^−1^ of white light and (e) 400 μmol m^−2^ s^−1^ at indicated wavelengths. In (e) photosystem II activity was assayed with O_2_ evolution (water to an artificial electron acceptor); in (a), (b), (d), and (e), the fluorescence parameter *F*_V_/*F*_M_ was used. In (a), the symbols show average *k*_PI_ values, based on at least three measurements, and error bars show SDs. In (b–e), fitting was done on the averaged data (of at least three independent experiments); error bars, drawn when larger than the symbol, show the SE of the fit. (f) Temperature dependence of production of carbon‐centered radicals, in the presence or absence of O_2_ (constant air or nitrogen bubbling), and in the presence or absence of DCMU, as indicated, by pumpkin thylakoids illuminated with white light, PPFD 2000 μmol m^−2^ s^−1^, in the presence of α‐(4‐pyridyl 1‐oxide)‐*N*‐*tert*‐butylnitrone (POBN). The reaction product of POBN and the radicals (POBN‐R adduct) was quantified by electron paramagnetic resonance. Closed bars represent the amount of the POBN‐R adduct before the light treatment (control). Each bar represents an average of at least three independent experiments, and error bars show SDs.

Besides UV illumination, anaerobicity is a condition where photoinhibition has been previously shown to occur even though ^1^O_2_ is not produced. Therefore, to better understand the connection between ^1^O_2_ production and photoinhibition, we next illuminated thylakoids in anaerobic conditions. In this case, photoinhibition was assayed with the *F*_V_/*F*_M_ fluorescence parameter. As shown by Sipka *et al*. (2021), *F*_V_/*F*_M_ cannot be taken as a measure of the PSII quantum yield but can be used as an empirical PSII activity parameter. The experiments showed an essentially similar temperature dependence of photoinhibition under anaerobic and aerobic conditions (Fig. 4a). The action spectrum, another characteristic of the reaction mechanism, was similar for anaerobic and aerobic photoinhibition (Fig. 4b).

Photoinhibition *in vitro* can be reversible under anaerobic conditions, mainly because of depletion and rebinding of bicarbonate to PSII (Sundby *et al*., 1992). However, we did not observe any reversibility, nor did addition of bicarbonate affect photoinhibition (Fig. S5).

To further test the effect of ^1^O_2_ production on photoinhibition, we illuminated pumpkin thylakoids in the presence of two efficient ^1^O_2_ scavengers, water‐soluble histidine and hydrophobic α‐tocopherol, and found no effect on photoinhibition or on its temperature dependence (Fig. 4c). In the dark, these compounds did not show any clear effects either, except small protection by histidine against light‐independent inactivation of PSII (Fig. S6). These results show that either photoinhibition is independent of ^1^O_2_ or has parallel mechanisms, some of them independent of O_2_.

We also measured aerobic and anaerobic photoinhibition in the presence of DCMU, a herbicide that blocks electron transfer from Q_A_ to Q_B_. Again, photoinhibition was measured with *F*_V_/*F*_M_. Interestingly, in the presence of DCMU, the temperature dependence was lost (Fig. 4d), and in the visible range the action spectrum was flatter than in the absence of DCMU (Fig. 4e). Owing to a relatively low resolution, we may have missed a previously observed peak at 670 nm (Santabarbara *et al*., 2001).

Photoinhibition proceeded faster in anaerobic than in aerobic conditions (Fig. 4b). To test whether accumulation of C‐centered radicals would explain this result, we illuminated pumpkin thylakoids in the presence of the radical probe POBN and found more radicals after anaerobic than after aerobic illumination (Fig. 4f). However, accumulation of radicals did not show a clear temperature dependence at 5–35°C in the absence of DCMU (Fig. 4f), contrary to photoinhibition. Both in the presence and absence of O_2_, DCMU strongly suppressed radical accumulation but, curiously, also imposed a positive temperature dependence (Fig. 4f). Accumulation of C‐centered radicals was negligible in the dark at 20°C, and the signal, obtained by illumination, remained stable in the dark at 5–35°C (Fig. S7).

A connection between misses and photoinhibition was further probed by comparing the pH dependences of the two phenomena. The *k*_PI_ value decreased by one‐third from pH 6.8 to pH 8.2 (Fig. S8), whereas misses show little pH dependence in this range (Messinger & Renger, 1994). However, Davletshina & Semin (2020) have suggested that the pH dependence of photoinhibition may reflect pH‐dependent changes in the structure of the OEC. These changes may affect photoinhibition without affecting the miss rate.

### Temperature of dark incubation affects fluorescence‐based estimations of photoinhibition

Finally, we assayed photoinhibition in pumpkin leaves both with *F*_V_/*F*_M_ and O_2_ evolution. A similar positive temperature dependence was obtained irrespective of the assay method (Fig. S9a). However, if the 30 min dark incubation before the *F*_V_/*F*_M_ measurement was conducted at 5°C (the illumination temperature), photoinhibition appeared to proceed faster than when the dark incubation was done at 22°C (Fig. S9b). Thus, dark‐incubation temperature may greatly affect the extent of the observed decline in *F*_V_/*F*_M_.

## Discussion

### Miss‐associated recombination of P_680_ ^+^ Q_A_ ^−^ is responsible for singlet oxygen production of photosystem II

In plants, chloroplasts are the most important producers of ^1^O_2_ in the light (Hideg *et al*., 2002; Prasad *et al*., 2018), and the potential of ^3^P_680_ for the ^1^O_2_ production has been clear for many years (Telfer *et al*., 1994). ^3^P_680_ is produced by charge recombination reactions, but the importance of different recombination pathways has not been known. It has been suggested that S_*n*_Q_A/B_^−^ → S_*n*−1_Q_A/B_ reactions produce enough ^3^P_680_ and subsequently ^1^O_2_ to inactivate PSII in weak light or during illumination with short laser pulses (Keren *et al*., 1997; Vass, 2011). The recombination of P_680_^+^Pheo^−^ has been suggested to be important in ^1^O_2_ production in strong light when Q_A_ is mostly reduced (Vass, 2011; Rehman *et al*., 2013). Our data show that these recombination reactions cannot significantly contribute to ^1^O_2_ production; the temperature dependences of the slow recombinations are too steep and that of the rapid recombination of the primary radical pair is too flat to account for the observed temperature dependence of ^1^O_2_ formation by thylakoid membranes (Figs 1, 2). Furthermore, ^3^P_680_ is short‐lived in the presence of Q_A_^−^ (Hillmann *et al*., 1995; Santabarbara *et al*., 2003).

**Fig. 5:**
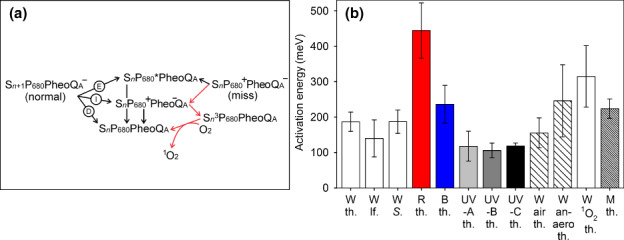
Activation energies and recombination pathways. (a) Recombination pathways of photosystem II (PSII). A charge separation produces either reduction of Q_A_ and advancement of the S‐state of oxygen (O_2_) evolving complex (normal) or reduction of Q_A_ without advancement of the S‐state (miss). The three charge recombination pathways from the ‘normal’ state are depicted as direct (D), indirect (I), and excitonic (E). The excited triplet state of the PSII reaction center Chl(s) ^3^P_680_ can be produced either from the indirect (via the primary charge pair P_680_^+^Pheo^−^) recombination pathway or equivalent recombination from the miss configuration, but the miss recombination (red arrows) is much faster and has a temperature dependence matching singlet O_2_ (^1^O_2_) production. (b) Activation energy of photoinhibition in pumpkin thylakoids (th.), pumpkin leaves (lf.), and *Synechocystis* cells (*S*.), obtained under illumination in white (W), blue (B), or red (R) light and in ultraviolet (UV)‐A, UV‐B, or UV‐C radiation, measured using O_2_ evolution as a PSII activity assay. Activation energy of photoinhibition in aerobic (air; as a control) and anaerobic (anaero) conditions, quantified using the fluorescence parameter *F*_V_/*F*_M_ as a PSII activity assay (sparsely hatched bars). Activation energy of ^1^O_2_ production, measured with a histidine‐based method. Activation energy of the miss factor of spinach thylakoids (M; densely hatched bars), calculated from published data (Isgandarova *et al*., 2003). Error bars show the SEs. Original data are from Figs 1, 2, 3, 4. The rates of the reactions have different units, but calculation of activation energies is independent of the unit. For the fits to the Arrhenius equation, see Figs 1, 3, 4.

The miss‐associated recombination of P_680_^+^Q_A_^−^ is the only reaction with a temperature response similar to the observed ^1^O_2_ production (Figs 1, 2), and therefore the data strongly suggest that ^1^O_2_ is produced in a reaction between O_2_ and ^3^P_680_, where ^3^P_680_ is formed by the miss‐associated recombination of P_680_^+^Q_A_^−^ (see Fig. 5a). This reaction, like all recombination reactions of PSII (except for the recombination of the primary pair), is expected to have a high triplet yield because of the lack of spin correlation of the reactants.

### Energetics of misses, recombination reactions, and singlet oxygen formation

The miss‐associated recombination of S_1_P_680_^+^Q_A_^−^ would obviously proceed via pathways equivalent to the reducing side of the indirect and excitonic pathways of S_2_Q_A_^−^ recombination (Rappaport & Lavergne, 2009); that is, via the S_1_P_680_^+^Pheo^−^Q_A_ intermediate. Assuming that the pre‐exponential factor *s* takes similar values in P_680_^+^Q_A_^−^ recombination as in S_2_Q_A_^−^ recombination (Table S1), and knowing that the Gibbs energy change from P_680_^+^PheoQ_A_^−^ to P_680_^+^Pheo^−^Q_A_ is 0.33 eV (Dau & Zaharieva, 2009), the time constant of the recombination reaction P_680_^+^PheoQ_A_^−^ → P_680_^+^Pheo^−^Q_A_ (which may lead to ^3^P_680_) would be 191 μs at 25°C (see Calculations in Methods S1), in agreement with the time constant of 100–200 μs, measured by Renger & Wolff (1976).

A miss of the OEC occurs because the S‐state does not always advance, not because the recombination of P_680_^+^Q_A_^−^ occasionally wins competition with normal advancement of the S‐state (Pham *et al*., 2019). The simplest mechanism by which misses can originate from the oxidizing side of PSII is that the OEC has a miss‐prone state (OEC_miss_), characterized by a slow S_*n*_ → S_*n*+1_ transition. Thus, the temperature dependence of ^1^O_2_ formation reflects the activation energies of a multistep reaction beginning with the reaction OEC_normal_ → OEC_miss_, which, based on analysis of the results of Isgandarova *et al*. (2003), has an *E*_a_ of 0.224 eV (Fig. 5b). This *E*_a_ is the enthalpy difference between the transition state of the reaction OEC_normal_ → OEC_miss_ and OEC_normal_. If we assume that the miss factor is 8% at 25°C and all misses occur in the S_2_ → S_3_ transition, then the equilibrium constant of the reaction OEC_normal_ → OEC_miss_ is 0.32. As $K_{\mathrm{eq}}=e^{-\Delta G_{r}/k_{b}T}$, where Δ*G*_r_ is the Gibbs energy change of the reaction, this further implies that OEC_miss_ is 0.03 eV above OEC_normal_.

After a charge separation and reduction of Q_A_, a multistep reaction forming ^1^O_2_ consists of the reaction OEC_normal_ → OEC_miss_, recombination P_680_^+^Q_A_^−^ → P_680_Q_A_, formation of ^3^P_680_, and a reaction between O_2_ and ^3^P_680_ (Fig. 5a). An effective *E*_a_ of a multistep reaction is calculated by adding *k*_b_ *T* and the sum of the standard‐state enthalpies (subtracting those of the reactants) of all intermediates and transition states of the reaction, where each enthalpy value is multiplied by its degree of rate control (DRC) (Mao & Campbell, 2019). The radical pair P_680_^+^Pheo^−^ represents the transition state of the P_680_^+^Q_A_^−^ → P_680_Q_A_ recombination reaction, which implies that the *E*_a_ of the recombination reaction is the same as the redox potential difference of the Pheo/Pheo^−^ and Q_A_/Q_A_^−^ pairs, 0.33 eV (Dau & Zaharieva, 2009). A slightly smaller *E*_a_ of ^1^O_2_ formation, 0.31 eV (Fig. 5b), indicates that contributions from the formation of ^3^P_680_ and the reaction between O_2_ and ^3^P_680_ to the *E*_a_ are close to zero. Indeed, formation of ^3^P_680_ occurs even at 10 K (Lendzian *et al*., 2003), indicating that the triplet formation does not depend on temperature at 2–35°C. ^1^O_2_ production by illuminated rose bengal, in turn, has an activation energy of only 0.055 eV (Fig. S2b). This *E*_a_ belongs to a complicated reaction in which the conversion of ^1^O_2_ to O_2_ via collision of H_2_O competes with the reaction of ^1^O_2_ with histidine. We now assume that the products DRC × *E*_a_ for the reactions between ^3^P_680_ and O_2_, followed by the reaction between ^1^O_2_ and histidine, are negligible because these reactions occur after ^1^O_2_ formation and because the *E*_a_ contribution of this series of steps would be comparable to the small *E*_a_ measured for the production and detection of ^1^O_2_ in the rose bengal system. With these assumptions, direct application of the model of Mao & Campbell (2019) for the measured *E*_a_ of ^1^O_2_ formation results in *E*_a_(^1^O_2_) = *k*_b_ *T* + DRC1 × 0.224 eV + DRC2 × 0.03 eV + DRC3 × 0.33 eV = 0.31 eV, where DRC1 belongs to the moving of the reaction coordinate from OEC_normal_ to the transition state of the reaction OEC_normal_ → OEC_miss_, DRC2 belongs to the formation of OEC_miss_ from the transition state, and DRC3 belongs to the transitional step P_680_^+^PheoQ_A_^−^ → P_680_^+^Pheo^−^Q_A_. We further assume that DRC2 is zero, and, taking into account that DRC1 + DRC2 + DRC3 = 1, we get DRC1 = 0.39 and DRC3 = 0.61 (calculations in Methods S1). Thus, the rate of ^1^O_2_ formation is controlled both by the frequency of misses and by the rate of the miss‐associated recombination reaction.

### Photoinhibition has three parallel mechanisms

Our data conclusively show that the temperature dependence of photoinhibition is positive both *in vivo* and *in vitro*. The result agrees with several earlier studies (Tyystjärvi *et al*., 1994; Lazarova *et al*., 2014; Ueno *et al*., 2016; Mattila *et al*., 2020) but contrasts with the findings of Tsonev & Hikosaka (2003) and Kornyeyev *et al*. (2003), who found a strong negative temperature dependence. The difference might suggest that, in our experiments, excitation pressure (suggested to be the cause of fast photoinhibition at low temperatures) was similar at all temperatures. Though this actually is true for the *in vitro* results (Fig. S3), the PSII yield of pumpkin leaves showed a clear negative dependence on temperature, indicating decreased excitation pressure at higher temperatures (Fig. 2c). Furthermore, no connection between excitation pressure and *k*_PI_ at different temperatures was found in our earlier study (Mattila *et al*., 2020). A possible reason why Tsonev & Hikosaka (2003) and Kornyeyev *et al*. (2003) observed a negative temperature dependence for photoinhibition is that they used the Chl fluorescence parameter *F*_V_/*F*_M_ for quantification of PSII activity. A low temperature during dark incubation between illumination and fluorescence measurement can affect the results by slowing the relaxation of nonphotochemical fluorescence quenching (Fig. S9); Tsonev & Hikosaka (2003) indeed performed the dark incubation and photoinhibition treatment at the same temperature.

^1^O_2_ has often been suggested to function as a causal agent of photoinhibition (Vass, 2011; Tyystjärvi, 2013), but the occurrence of photoinhibition under UV radiation and in anaerobic conditions where ^1^O_2_ is not formed indicates that parallel mechanisms must function. We will first treat the visible‐light‐specific photoinhibition mechanisms as one combined mechanism functioning parallel with another mechanism that is fully responsible for photoinhibition under UV radiation. The apparent *E*_a_ of photoinhibition, in the presence of two parallel pathways, is the weighted sum, calculated as *E*_a_(total) = (*k*_1_ *E*_1_ + *k*_2_ *E*_2_)/(*k*_1_ + *k*_2_), where *k*_*i*_ and *E*_*i*_ are the rate constant and *E*_a_ of reaction *i* (*i* = 1, 2), respectively. As *k*_PI_ is proportional to photon flux density (Tyystjärvi & Aro, 1996), we can simplify the equation by the normalization *k*_1_ + *k*_2_ = 1.

We assume that the UV mechanism is triggered by light absorption by the Mn ions of OEC (Hakala *et al*., 2005) and first calculate the contribution of the Mn mechanism in visible light. Absorbance values of Mn complexes decrease with wavelength (e.g. Horner *et al*., 1999), and the contribution of the Mn mechanism is likely to be negligible in long‐wavelength (red) visible light (Hakala *et al*., 2005; Ohnishi *et al*., 2005). We therefore postulate that photoinhibition in red light is entirely caused by mechanism(s) dependent on the light absorption by Chls, whose combination consequently must have an apparent *E*_a_ of 0.46 eV (Fig. 5b). If photoinhibition of thylakoids in white light (*E*_a_ 0.20 eV) is a linear combination of the UV mechanism (*E*_a_ 0.12 eV in UV‐A) and the mechanism(s) functioning in red light, then the Mn mechanism contributes 76% in white and 65% in blue light. For more details, see Calculations in Methods S1. Such a high contribution of a mechanism that is independent of Chl absorption agrees with the relatively small protective effect of nonphotochemical quenching of Chl excitations (Sarvikas *et al*., 2006; Havurinne & Tyystjärvi, 2017), although it can also be explained by assuming involvement of uncoupled Chls in photoinhibition (Santabarbara *et al*., 2001). The mechanistic details of the Mn mechanism, except for the release of Mn ions from OEC (Hakala *et al*., 2005), are still obscure, and therefore a dependence from O_2_ or involvement of reactive O_2_ species in the Mn mechanism cannot be excluded. However, photoinhibition induced by UV‐A radiation was only weakly affected by removal of O_2_ (Fig. 4b).

The fact that visible light induces photoinhibition in anaerobic conditions indicates that a ^1^O_2_‐independent mechanism must exist. The Mn mechanism cannot explain all such photoinhibition because the *E*_a_ of anaerobic photoinhibition in visible light is 0.26 eV whereas that of the UV‐active mechanism is 0.12 eV. The effect of O_2_ on the rate of photoinhibition in UV radiation and visible light can be used to estimate the importance of the Mn mechanism in anaerobic conditions in visible light. The ratio *k*_PI_(anaerobic)/*k*_PI_(aerobic) is, on average, 3.45 in visible light but only 1.2 at 312 nm (Fig. 4b), suggesting that anaerobicity does not boost the Mn mechanism and thus that all increase in the rate of photoinhibition due to lack of O_2_ is accounted by an O_2_‐independent visible‐light‐specific mechanism. Following this assumption, the Mn mechanism, accounting for 76% of visible‐light photoinhibition in aerobic conditions, only contributes by 22% in anaerobic conditions. Now, the *E*_a_ of the O_2_‐independent visible‐light mechanism, functioning in parallel to the Mn mechanism, becomes (0.26 eV − 0.22 × 0.12 eV)/0.78 = 0.30 eV, which is somewhat but not drastically higher than the *E*_a_ of the misses (see Calculations in Methods S1), suggesting a causal relationship between misses and the O_2_‐independent mechanism. We suggest that the O_2_‐independent mechanism is the classical donor‐side photoinhibition (Callahan & Cheniae, 1985; Chen *et al*., 1992; Jegerschöld & Styring, 1996), in which P_680_^+^, if long‐lived, commits a harmful oxidation in PSII. Misses, by prolonging the lifetime of P_680_^+^ by barring electron flow from OEC, may trigger this reaction in healthy PSII. If the miss‐associated recombination has a time constant of 191 μs while electron transfer from Q_A_^−^ to Q_B_ takes 500 μs, then the miss mechanism would prolong the lifetime of P_680_^+^ in 38% of the cases because electron transfer from Q_A_^−^ to Q_B_ occurs before the miss‐associated P_680_^+^Q_A_^−^ recombination. Thus, electron transfer from Q_A_^−^ to Q_B_ after a miss would lock PSII to the P_680_^+^ state until the missed OEC finally advances the S‐state.

The enhanced formation of C‐centered radicals in anaerobic conditions (Fig. 4f) may suggest that oxidation of PSII proteins by P_680_^+^ is linked to anaerobic photoinhibition. In line with this suggestion, DCMU strongly suppressed radical formation. However, anaerobic photoinhibition is not suppressed by DCMU, indicating that not all protein oxidation events lead to loss of PSII activity; DCMU may alter the probabilities of different oxidation events. The lack of matching temperature dependence in radical formation and photoinhibition responses (Fig. 4d,f) confirms that the relationship between photoinhibition and formation of protein radicals is not straightforward.

The relative rates of the ^1^O_2_‐dependent and O_2_‐independent visible‐light‐specific mechanisms in the presence of O_2_ cannot be estimated from the present data, but the *E*_a_ of their combination, 0.46 eV, and that of the O_2_‐independent reaction, 0.30 eV, imply that the *E*_a_ of the ^1^O_2_‐dependent mechanism is ≥ 0.46 eV. The strong photoprotective effect of carotenoids (Jahns *et al*., 2000; Hakkila *et al*., 2013) suggests that the ^1^O_2_‐dependent mechanism is the major contributor among the Chl‐dependent mechanisms. Owing to the high *E*_a_ value of ^1^O_2_‐dependent photoinhibition, the relative contribution of this mechanism is expected to increase with temperature. This may explain why studies with cyanobacteria and algae that are cultivated and treated with high light in their cultivation temperature often yield results supporting the importance of ^1^O_2_ (Jahns *et al*., 2000; Fufezan *et al*., 2007; Hakkila *et al*., 2013; Treves *et al*., 2016), whereas the results of the present study (mostly conducted on plant thylakoids) suggest a large contribution of the Mn mechanism that has a low *E*_a_.

Temperature dependence may not exactly reflect *E*_a_ for PSII charge recombination reactions with tunneling character (Moser *et al*., 2005). The good fit of the thermoluminescence data (Fig. 2a) may suggest that *E*_a_ is not damped, but the accuracy of the photoinhibition data does not allow estimation of dampening of the activation. Furthermore, we cannot exclude the possibility that the matching temperature dependences are fortuitous. In particular, ^1^O_2_ formation by uncoupled Chls (Santabarbara *et al*., 2001, 2002, 2003) might have a temperature dependence matching that measured for ^1^O_2_ (Fig. 1).

## Author contributions

ET designed the research; HM and SM performed the research; ET and HM analyzed the data; HM, ET and TT wrote the paper with contribution from SM.

## Supporting information


**Fig. S1** Energy spectra of light sources used in the present study.
**Fig. S2** Temperature dependence of the histidine method.
**Fig. S3** Effect of temperature on the yield of photosystem II electron transfer.
**Fig. S4** Measurements of photoinhibition of photosystem II.
**Fig. S5** Photoinhibition under anaerobic conditions, in the absence or presence of sodium bicarbonate.
**Fig. S6** Temperature dependences of dark inactivation in the absence and presence of histidine or in the absence and presence of α‐tocopherol.
**Fig. S7** Detection of carbon‐centered radicals from pumpkin thylakoids with α‐(4‐pyridyl 1‐oxide)‐*N*‐*tert*‐butylnitrone.
**Fig. S8** pH dependence of photoinhibition.
**Fig. S9** Comparison of fluorescence and oxygen evolution assays for quantification of photoinhibition.
**Methods S1** Calculations.
**Table S1** Parameters obtained from the thermoluminescence measurements.
Please note: Wiley is not responsible for the content or functionality of any Supporting Information supplied by the authors. Any queries (other than missing material) should be directed to the *New Phytologist* Central Office.

## Data Availability

Raw data are available in Mendeley Data (https://data.mendeley.com/datasets/sg4bbmnjvc/1).
