# Supplementary material for: Singlet oxygen production by photosystem II is caused by misses of the oxygen evolving complex
Source: New Phytol. 2022 Oct 14;237(1):113–25. doi: 10.1111/nph.18514 (PMC10092662; doi:10.1111/nph.18514)
Supplement: Supplementary file 1 — Fig. S1 Energy spectra of light sources used in the present study. Fig. S2 Temperature dependence of the histidine method. Fig. S3 Effect of temperature on the yield of photosystem II electron transfer. Fig. S4 Measurements of photoinhibition of photosystem II. Fig. S5 Photoinhibition under anaerobic conditions, in the absence or presence of sodium bicarbonate. Fig. S6 Temperature dependences of dark inactivation in the absence and presence of histidine or in the absence and presence of α‐tocopherol. Fig. S7 Detection of carbon‐centered radicals from pumpkin thylakoids with α‐(4‐pyridyl 1‐oxide)‐N‐tert‐butylnitrone. Fig. S8 pH dependence of photoinhibition. Fig. S9 Comparison of fluorescence and oxygen evolution assays for quantification of photoinhibition. Methods S1 Calculations. Table S1 Parameters obtained from the thermoluminescence measurements. Please note: Wiley is not responsible for the content or functionality of any Supporting Information supplied by the authors. Any queries (other than missing material) should be directed to the New Phytologist Central Office. [file NPH-237-113-s001.pdf]

New
Phytologist

New Phytologist Supporting Information

Article title: Singlet oxygen production by Photosystem II is caused by misses of the oxygen evolving complex
Authors: Heta Mattila, Sujata Mishra, Taina Tyystjärvi, Esa Tyystjärvi
Article acceptance date: 10 September 2022

The following Supporting Information is available for this article:

Fig. S1 Energy spectra of light sources used in the present study.

Fig. S2 Temperature dependence of the histidine method.

Fig. S3 Effect of temperature on the yield of PSII electron transfer.

Fig. S4 Measurements of photoinhibition of PSII.

Fig. S5 Photoinhibition under anaerobic conditions, in the absence presence of Na-bicarbonate.

Fig. S6 Temperature dependences of dark-inactivation in the absence and presence of histidine or in the absence and presence of α-tocopherol.

Fig. S7 Detection of carbon-centered radicals from pumpkin thylakoids with POBN.

Fig. S8 pH dependence of photoinhibition.

Fig. S9 Comparison of fluorescence and oxygen evolution assays for quantification of photoinhibition.

Table S1 Parameters obtained from the thermoluminescence measurements.

Methods S1 Calculations.

Fig. S1 Energy spectra of light sources used in the present study. (a) White light obtained with a slide projector equipped with a low voltage halogen lamp (continuous line), 10 W cold white LED (short-dashed line) and a 1000 W high pressure Xenon lamp (dashed line). (b) Blue (continuous line) and red (dashed line) light obtained with the Xenon lamp in combination of short- and long-pass filters. All spectra are normalized to their maximum value at the visible light range (400–700 nm).

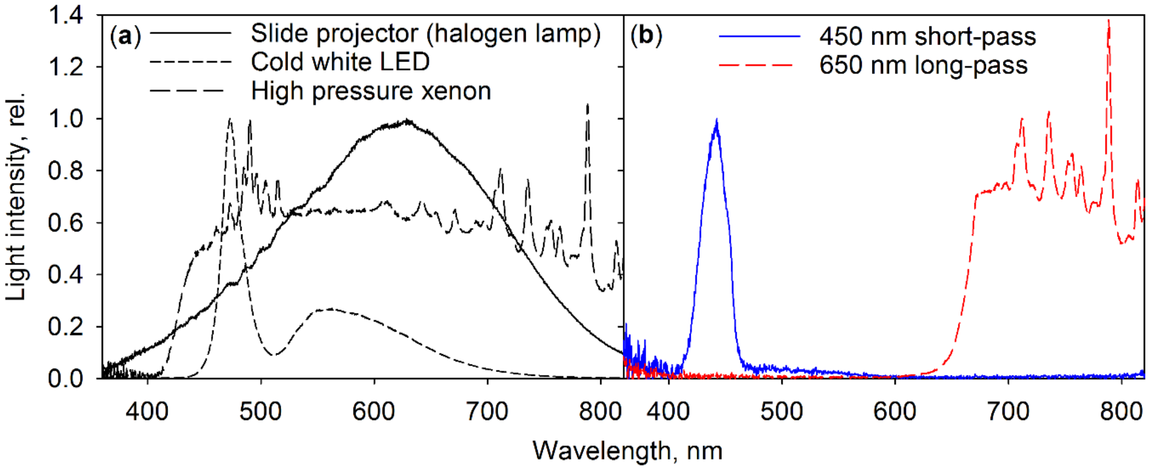

Fig. S2 Temperature dependence of oxygen consumption by illuminated (PPFD 2000 µmol $m^{-2}s^{-1}$) 1 µM Rose Bengal solution in the presence of 20 mM histidine, reflecting $^1O_2$ production. Each data point represents an average of three independent experiments. Error bars, drawn when larger than the symbol, show SD. (b) Calculation of the activation energy according to the Arrhenius equation, from the data in (a). The dashed line shows a linear fit to the data, revealing an Ea of 0.055 eV.

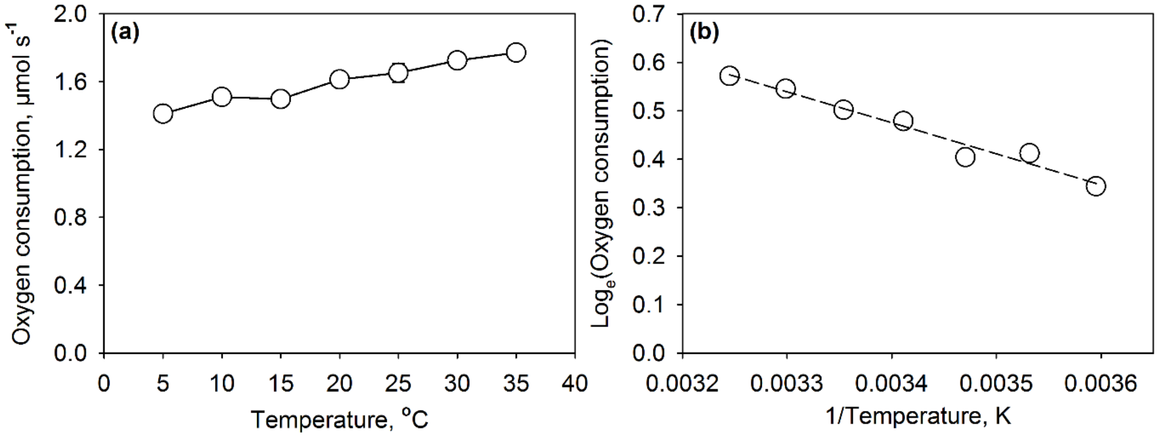

Fig. S3 Effect of temperature on the yield of PSII electron transfer in isolated pumpkin thylakoid membranes (dark grey circles), pumpkin leaves (triangles) and *Synechocystis* cells (squares). $(F_M'-F)/F_M'$ was measured after 1 min (thylakoids), 15 (white symbols), 30 (light grey symbols) and 45 min (black symbols) of white light illumination, PPFD 1500 µmol $m^{-2}s^{-1}$ (thylakoids and leaves) or 750 µmol $m^{-2}s^{-1}$ (*Synechocystis*), at the specified temperatures. Each data point represents an average of three independent measurements. Error bars, drawn when larger than the symbol, show SD.

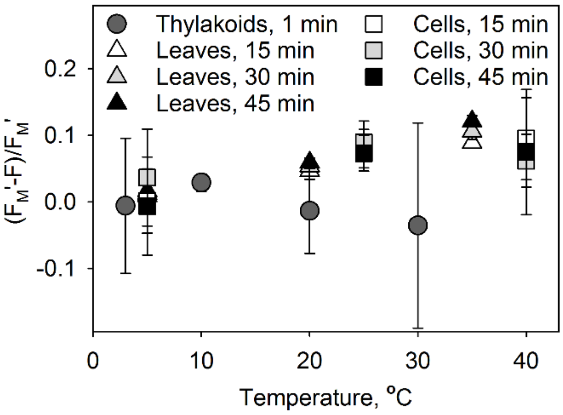

Fig. S4 Photoinhibition of PSII. (a) An example of a photoinhibition experiment; a decrease in the rate of light-saturated oxygen evolution of PSII (H_2O to DMBQ), measured at 22 °C from aliquots of the treated sample (isolated pumpkin thylakoids illuminated with white light, PPFD 1500 µmol $m^{-2}s^{-1}$, or incubated in darkness, at 25 °C). The dashed lines show the best fits to a first-order reaction equation, based on which a rate constant of photoinhibition (k_{PI}) was calculated. Each data point shows an average of three independent experiments. Error bars, drawn when larger than the symbol, show SD. (b) Temperature dependence of photoinhibition in pumpkin thylakoids, illuminated as described in (a) at the indicated temperatures. Individual time courses of photoinhibition or dark-inactivation were fitted to the first-order reaction equation; the symbols show average k_{PI} values from at least three individual measurements, and the error bars, drawn when larger than the symbol, show the SD. The rate constants of dark inactivation (solid circles) were subtracted from raw rate constants of photoinhibition (open circles) to obtain the final k_{PI} values (Light-Dark; triangles). (c) Temperature dependence of photoinhibition in lincomycin-treated pumpkin leaves and *Synechocystis* cells, illuminated

with the PPFDs of 1500 µmol $m^{-2}s^{-1}$ and 750 µmol $m^{-2}s^{-1}$, respectively. Prior to the oxygen evolution measurement and after an illumination, thylakoids were isolated from the pumpkin leaves. In the case of leaves and cells, dark inactivation was not measured, but otherwise the samples were treated as in (a–b), except that in the case of *Synechocystis* cells, oxygen evolution was measured at 32 °C, DCBQ used as an electron acceptor. Oxygen evolution rates of the controls (before a treatment) were 366 ± 68 (thylakoids) and 284 ± 22 (leaves; thylakoids isolated before and after treatments) µmol O_2 (mg chlorophyll)$^{-1}$ h^{-1}, and 1.44 ± 0.39 µmol O_2 (optical density at 730 = 1)$^{-1}$ h^{-1} for intact *Synechocystis* cells.

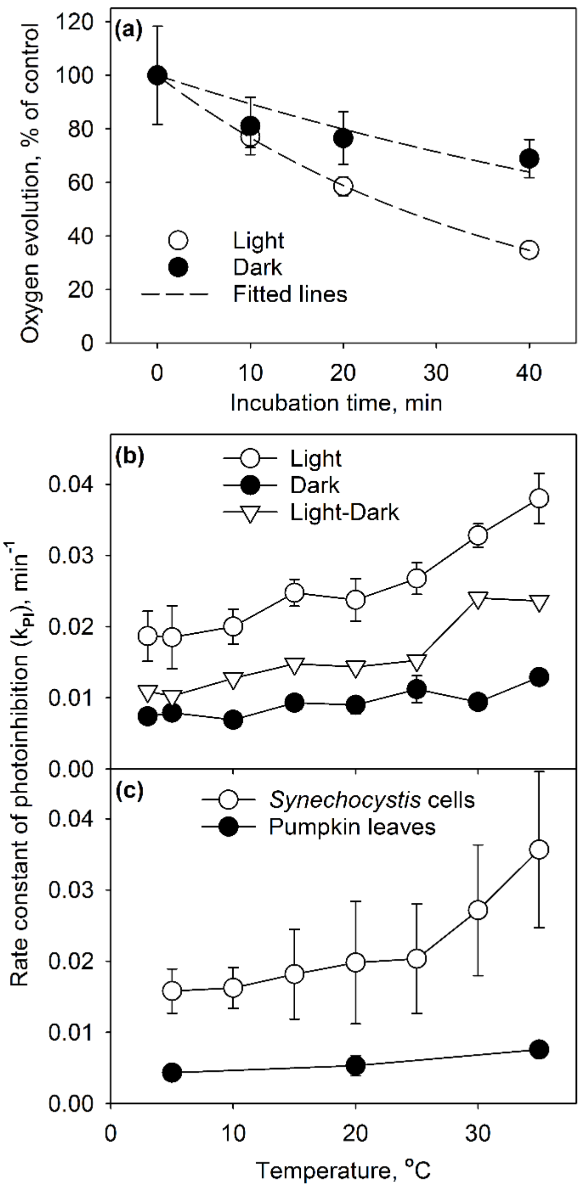

Fig. S5 Photoinhibition in pumpkin thylakoids under anaerobic conditions, in the absence (solid circles) or presence of 20 mM Na-bicarbonate (open circles). Thylakoids were illuminated (PPFD 2000 µmol $m^{-2}s^{-1}$) at 20 °C for 30 min (indicated with the yellow bar) with constant nitrogen bubbling, after which they were let to recover in room light for 15 min (grey bar) under ambient air with slow mixing. Light saturated rate of oxygen evolution of PSII (H_2O to DMBQ) was measured at 22 °C. Averages from three independent experiments are shown. Error bars, drawn when larger than the symbol, show SD.

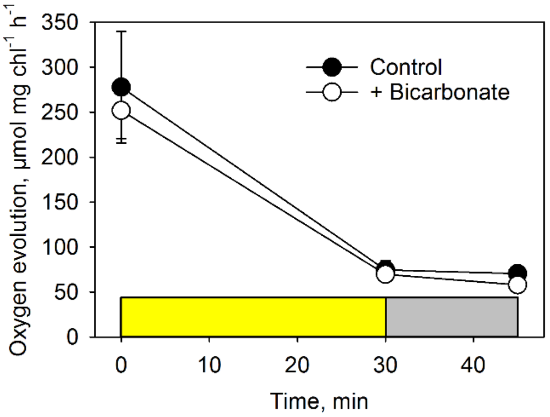

Fig. S6 Temperature dependences of dark-inactivation of pumpkin thylakoids, (a) in the absence (open diamonds) and presence of 5 mM histidine (solid diamonds) or (b) in the absence (open upward triangles) and presence of 0.5 mM α-tocopherol (solid downward triangles). PSII activity was assayed as the rate of light-saturated oxygen evolution (H_2O to DMBQ). Averages from at least three individual measurements have been used in the fitting, to obtain rate constants, and the error bars, drawn when larger than the symbol, show the SE of the fit. The experiments with histidine and tocopherol were done with different thylakoid batches.

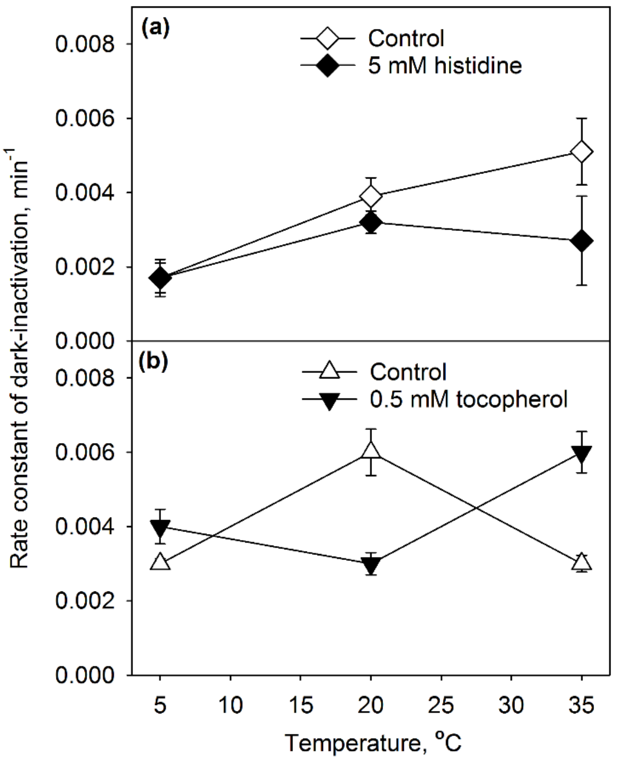

Fig. S7 Detection of carbon-centered radicals from pumpkin thylakoids with POBN. (a) Examples of the EPR signal of the sample before (Control) and after a 30 min illumination with white light (PPFD 2000 µmol $m^{-2}s^{-1}$) at 20 °C in the presence (Air) or absence (N_2) of oxygen, and in the absence or presence of DCMU. All lines except Control have been shifted upwards to facilitate comparison. (b) Amounts of carbon-centered radicals (POBN-R adduct) before (0 min) and after a 30 min aerobic or anaerobic incubation in the dark at 20 °C. (c) Amounts of carbon-centered radicals (POBN-R adduct) before (Ctl) and after a 10 min anaerobic illumination (PPFD 2000 µmol $m^{-2}s^{-1}$; Light 10 min N_2), after subsequent 15–30 min in the dark under nitrogen bubbling (Dark 15 or 30 min N_2) and after subsequent 15–30 min in the dark under air bubbling (Dark 15 or 30 min Air). The signals were quantified by the height of the first positive peak at 334.5–334.8 mT of the EPR signal; each bar represents an average of three independent experiments and the error bars show SD (b–c).

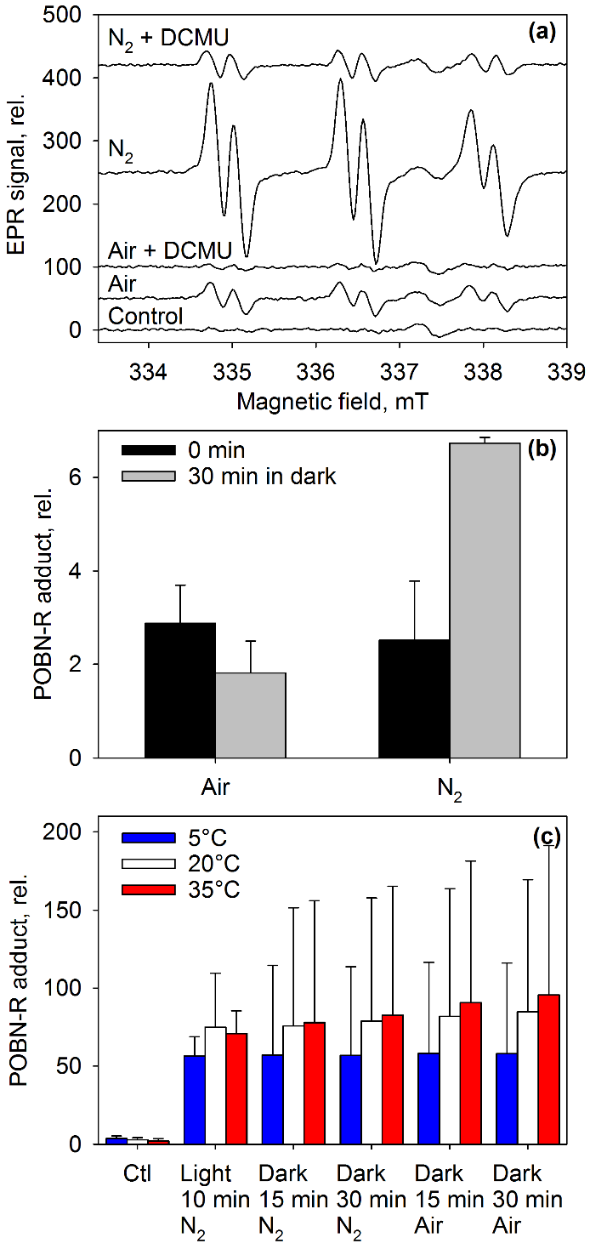

Fig. S8 pH dependence of photoinhibition. Pumpkin thylakoids were illuminated with white light (PPFD 1500 µmol $m^{-2}s^{-1}$) or incubated in darkness in photoinhibition buffer, pH adjusted as indicated, at 20 °C. The rate constants of photoinhibition (k_{PI}; Light - Dark) and dark-inactivation (k_{DARK}) were obtained by measuring three time series of photoinhibition, fitting each time series to the first-order reaction equation and averaging the results. Error bars show SD.

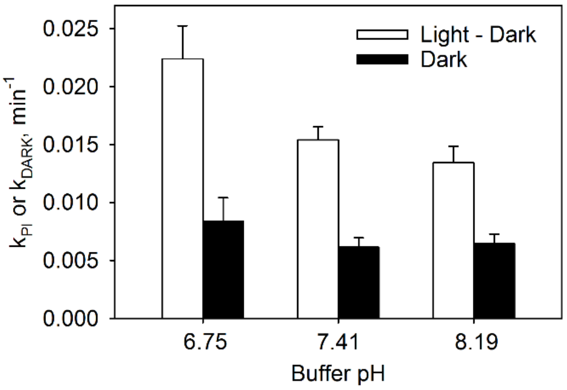

Fig. S9 Comparison of fluorescence and oxygen evolution assays for quantification of photoinhibition. (a) Pumpkin leaves, in the presence of 0.4 mg/ml lincomycin, were illuminated for 45 min with white light (PPFD 1500 µmol $m^{-2}s^{-1}$) at the indicated temperatures. The fluorescence parameter F_V/F_M was measured after 30 min in darkness at room temperature (22 °C) after which thylakoids were isolated and the light-saturated rate of oxygen evolution of PSII (H_2O to DMBQ) was measured. (b) Leaves, in the presence of lincomycin, were illuminated for 70 min at 5 °C. The fluorescence parameter F_V/F_M was measured after 30 min in darkness; the dark incubation was performed either at 5 °C or at room temperature (22 °C), as indicated. Each bar represents an average of three independent experiments and the error bars show SD.

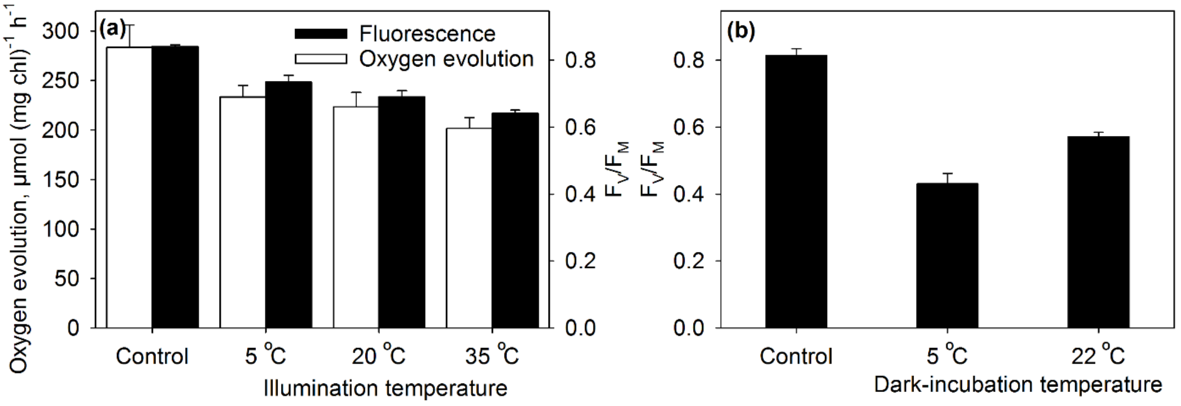

Table S1 Activation energy (Ea) and pre-exponential factor (s) obtained from the thermoluminescence measurements (Fig. 2a) and used to calculate the temperature dependencies of the charge recombination reactions (Fig. 2b).

Recombining species	$S_2Q_A^-$	$S_2Q_A^-$	$S_2Q_A^-$	$S_2Q_B^-$
Name of reaction	Indirect	Direct	Excitonic	
Ea, eV	0.60	0.32	0.88	0.71
s, s^{-1}	$2x10^9$	$1x10^4$	$3x10^9$	$1.8x10^{10}$

Methods S1 Calculations.

Calculation of the time constant of miss recombination

Let

$k = s_0$ x exp $(-Ea/k_bT)$ (Arrhenius's equation).

Assign

Ea = 330 meV,

$s_0 = 2 \times 10^9 s^{-1}$, and

T = 298 K.

Now, $k = 2 \times 10^9 s^{-1} \times \exp(-330 \text{ meV}/(0.086173 \text{ meV/K} \times 298 \text{ K})) = 5248.631 s^{-1}$, hence 1/k = 191 µs.

Calculation of the DCR values of steps of singlet oxygen production

Let

$Ea(tot) = k_bT + DRC1 \times H1 + DRC3 \times H3$,

where

Ea(tot) = 314.811 eV (the observed activation energy of $^1O_2$ formation),

k_b = Boltzmann's constant, 0.086173 meV/K,

T = 298 K,

DRC1 is the degree of rate control by the formation of the miss-prone state ($OEC_{normal} \rightarrow OEC_{miss}$),

H1 = 224 meV (enthalpy of the transition state of the formation of the miss-prone state; PSII with Q_A^- and OEC_{normal} is the zero level),

DRC3 is the degree of rate control by the miss-associated recombination reaction, and

H3 = 330 meV (enthalpy of the $P_{680}^{+}Pheo^{-}$ state, with the same zero level as above).

As DRC1 + DRC3 = 1,

we get (at 298 K)

DRC1 = (Ea(tot) - H2 - k_bT)/(H1 - H2) = (314.811 meV - 330 meV - 0.086173 meV/K x 298 K)/(224 meV - 330 meV) = 0.386, hence DRC2 = 0.614.

Calculation of the contribution of the Mn mechanism in visible light

Let

Ea(total) = $(k_1E_1 + k_2E_2)/(k_1 + k_2)$

Assign E_1 and E_2 from Fig. 5:

E_1 = 458 meV (Ea of the combination of the visible-light mechanisms, measured in red light where the Mn mechanism is negligible), and

E_2 = 119 meV (Ea of the Mn mechanism in UVA).

Ea(total) = 200 meV (Ea of photoinhibition in white light where both mechanisms function),

k_1 = rate constant of the Mn mechanism in visible light with such intensity that the rate constant of photoinhibition is 1, and

k_2 = combined rate constant of the visible-light mechanisms in the same conditions, thus $k_2 = 1 - k_1$.

Now, we get

200 meV = k_1 x 119 meV + (1 - k_1) x 458 meV, hence k_1 = 0.761, and k_2 = 0.239.

Calculation of the contribution of the Mn mechanism in visible light in anaerobic conditions

Use the earlier result showing that the Mn mechanism contributes by 76 % to photoinhibition in visible light in aerobic conditions.

Photoinhibition is 3.45 as fast in anaerobic as in aerobic conditions (calculated by comparing the average of visible-light data points in anaerobic and aerobic conditions from Fig. 4b).

Now, let the sum of the rate constants of the Mn mechanism (k_1) and the combination of the visible-light specific mechanisms (k_2) be $k_1 + k_2 = 1$ in aerobic conditions. In anaerobic conditions $k_1 + k_2$(anaerobic) = 3.45, hence k_1 = 0.76/3.45 = 0.22.

Calculation of the Ea of the oxygen-independent visible-light specific mechanism

Assume

$Ea(total) = (k_1E_1 + k_2E_2)/(k_1 + k_2)$.

Let

$k_1 + k_2 = 1$, hence $k_2 = 1 - k_1$,

where

$k_1 = 0.22$ (relative rate constant of Mn mechanism in anaerobic conditions in visible light),

$k_2 = 0.78$ (relative rate constant of the combination of visible-light specific mechanisms in anaerobic conditions),

$E_1 = 119$ meV (Ea of the Mn mechanism),

E_2 is the Ea of the oxygen-independent visible-light-specific photoinhibition, and

Ea(total) = 262 meV (Ea of photoinhibition in visible light in anaerobic conditions).

Now, $Ea(total) = k_1E_1 + (1 - k_1)E_2$,

hence $E_2 = (Ea(total) - k_1E_1)/(1 - k_1) = (262\ \text{meV} - 0.22 \times 119\ \text{meV})/0.78 = 302\ \text{meV}\ (0.30\ \text{eV})$.
